# Screening of Anaesthetics in Adult Zebrafish (*Danio rerio*) for the Induction of Euthanasia by Overdose

**DOI:** 10.3390/biology10111133

**Published:** 2021-11-04

**Authors:** Kristine von Krogh, Joseph Higgins, Yolanda Saavedra Torres, Jean-Philippe Mocho

**Affiliations:** 1 von Krogh Consult, 1266 Oslo, Norway; kristine.von.krogh@gmail.com; 2 The Francis Crick Institute, London NW1 1AT, UK; joe.higgins@crick.ac.uk (J.H.); Yolanda.Saavedra@crick.ac.uk (Y.S.T.); 3 Joint Production System Ltd., Potters Bar EN6 3DD, UK

**Keywords:** zebrafish, *Danio rerio*, euthanasia, anaesthesia, lidocaine, tricaine, benzocaine, overdose, reflexes, welfare

## Abstract

**Simple Summary:**

Although zebrafish are used in vast numbers in laboratories all over the world, no consensus has been reached in the scientific community on a humane, consistent, and effective method for euthanasia of this species. Here, we screened commonly used anaesthetic drugs to see if an overdose could induce loss of reflexes of adult zebrafish in a rapid and reliable manner, and without causing distress. The tested anaesthetics were isoeugenol, clove oil, 2-phenoxyethanol, tricaine, benzocaine, lidocaine hydrochloride, and etomidate. We found that lidocaine hydrochloride, buffered with sodium bicarbonate and ethanol to increase its efficacy, induces loss of reflexes in a fast, predictable, and relatively peaceful manner. We recommend its use for adult zebrafish euthanasia.

**Abstract:**

Zebrafish are often euthanized by overdose of anaesthesia. However, fish may have aversion towards some anaesthetics, and protocol efficacy varies between species. Using wild type adult *Danio rerio*, we assessed time to loss of opercular beat, righting, and startle reflexes during induction of anaesthetic overdose by either tricaine (0.5 g/L or 1 g/L), benzocaine (1 g/L), 2-phenoxyethanol (3 mL/L), clove oil (0.1%), isoeugenol (540 mg/L), lidocaine hydrochloride (1 g/L), or etomidate (50 mg/L). Initial screening demonstrated that benzocaine and buffered lidocaine hydrochloride achieved the fastest loss of reflexes. The rapid induction times were confirmed when retesting using larger batches of fish. The fastest induction was obtained with 1 g/L lidocaine hydrochloride buffered with 2 g/L NaHCO_3_, in which all adult zebrafish lost reflexes in less than 2 min. Next, we monitored signs of distress during benzocaine or buffered lidocaine hydrochloride overdose induction. The results indicated that buffered lidocaine hydrochloride caused significantly less aversive behaviors than benzocaine. Finally, we tested several buffers to refine the lidocaine hydrochloride immersion. The most efficient buffer for euthanasia induction using 1g/L lidocaine hydrochloride was 2 g/L NaHCO_3_ with 50 mL/L 96% ethanol, inducing immobility in less than 10 s and with only 2% of adult zebrafish displaying aversive behaviors during treatment.

## 1. Introduction

On welfare grounds or due to animals reaching experimental endpoints, fish are euthanized every day in the laboratories. Directive 2010/63/EU [1], which covers the protection of animals used for scientific purposes, states that fish should be euthanized either through anaesthetic overdose, concussion/percussive blow to the head, or electrical stunning. To ensure cessation of brain activity and blood circulation, euthanasia should be completed with a second procedure, e.g., exsanguination, pithing, maceration, or decapitation. The method of choice should reflect the circumstance of why the fish is to be euthanized in the first place. For instance, if the body tissue is to be used for further experimental analysis, some physical methods and some anaesthetic compounds may not be suitable, as they may damage cells or affect physiological and experimental data [2,3,4]. Additional factors such as safety for personnel, operator’s experience, cost, and environmental impact should also be taken into consideration when deciding upon the optimal euthanasia protocol. However, fish experience, i.e., perception of the event and welfare during the process, remains the key question to address.

Zebrafish (*Danio rerio*) is extensively used as a teleost model species in hundreds of research laboratories worldwide with several hundred thousand fish being euthanized every year [5,6]. The most common method of zebrafish euthanasia is an overdose of tricaine (ethyl 3-aminobenzoate methanesulfate; also known as MS-222, TMS, Metacaine, Finquel) [7]. However, there have been alarming reports of the drug triggering aversive behaviors during euthanasia induction in zebrafish (e.g., piping, twitching, erratic swimming, and increased activity) [8,9,10]. These behaviors may affect as many as 30–90% of animals [8,9], but the severity of the aversion may depend on the time of the day [10]. Furthermore, preference and conditioned place-avoidance tests revealed aversion even for lower doses of tricaine [11,12]. Alternative methods to euthanasia by tricaine overdose should, therefore, be considered for zebrafish [7].

When deciding upon the best method for euthanasia, the species at hand must be taken into consideration, as different fish species may have vastly different morphology, size, and physiology. *Danio rerio* is a small freshwater species and may not be susceptible to the same treatments as, for instance, a large marine species. This is not only true for physical methods but includes anaesthetic overdoses, as anaesthetic agents may cause differential effects in different species [13,14,15]. For instance, while 0.4 g/L lidocaine hydrochloride (HCl) irreversibly euthanized adult zebrafish [9], doses up to 0.8 g/L induced anaesthesia with full recovery in the similarly sized adult marine medaka (*Oryzias dancena*) [16]. Moreover, the efficiency of a compound may be related to the age and size of the fish [17]. For instance, while lidocaine HCl treatment effectively euthanized adult zebrafish, as described above, the compound was not able to induce cardiac arrest in larval zebrafish, even at higher doses (up to 1 g/L) of exposure [9].

Animal welfare is a key aspect to an optimal euthanasia protocol. As mentioned above, zebrafish can display signs of discomfort and stress during anaesthesia or euthanasia induction, including with the most used anaesthetic, tricaine, or with alternative methods such as hypothermic shock [8,18,19]. Studies have also demonstrated that zebrafish experience some anaesthetics as aversive even at lower doses of exposure [11,12]. Therefore, it is advisable that the chosen euthanasia procedure induces the least distress possible and that suitable species- and age- specific protocols are developed.

The purpose of the experiments described here was to reduce the distress of adult zebrafish during induction of euthanasia by overdose of anaesthesia. To achieve this, we aimed to identify an anaesthetic solution that would inhibit reflexes as fast as possible, with the least observable distress, and in the most reliable manner. To challenge reliability of anaesthetic protocols, we considered reflex inhibition for 100% of tested animals, and we reproduced conditions reflecting routine husbandry practice. Thus, anaesthetic screenings were performed with varying numbers of fish per batch, and anaesthetic baths were reused throughout the working day. First, we compared induction time to loss of reflexes for seven common anaesthetics: isoeugenol, clove oil (eugenol), 2-phenoxyethanol (2-PE), tricaine, benzocaine, lidocaine HCl, and etomidate [7]. The most efficient compounds from the initial screening were further assessed regarding efficiency on larger batches and disturbance of fish behavior during exposure. Finally, impact of different buffers on fish behavior and induction time to loss of reflexes were measured.

## 2. Materials and Methods

### 2.1. Animals and Housing

Zebrafish (*Danio rerio*) were kept in the animal facilities of the Francis Crick Institute, London, UK. The fish were housed in a recirculating system (Tecniplast, Buguggiate, Italy) supplied by Reverse Osmosis (RO) water. The 10,000 L of water were filtered with ultraviolet light, carbon filtration and maintained as follows: pH 7.6, conductivity 500 µS, 28.0 °C, 3 degrees of hardness, 2 degrees of alkalinity, 10% daily water renewal, 14:10 light/dark cycle, with dosing towers for salt (Coral Pro Salt, Red Sea, USA) and buffer (sodium hydrogen carbonate, Fisher Chemical, Loughborough, UK). The animals received *Artemia salina nauplii* (ZM Brine Shrimp Cysts Premium 240 Grade, Zebrafish Management Ltd., Winchester, UK) and SAFE caviar 300–500 (SAFE, Augy, France) daily. A total of 1409 adult fish (aged 7 to 27 months post hatching) of both sexes and of ten strains were included in the experiments. First generation progeny from the Zebrafish International Resource Center (ZIRC, Eugene, OR, USA) were used for four strains: AB, TU, TL, and NHGR-1. First generation progeny from the European Zebrafish Resource Center (EZRC, Eggenstein-Leopoldshafen, Germany) were used for three strains: AB, TU, and TL. And three strains were homebred for many generations: London AB, LIF TU, and Lewis. All fish were kept and used in compliance with the Directive 2010/63/EU and the United Kingdom’s Animals (Scientific Procedures) Act (The Francis Crick Institute, London, UK, approval X49D044E1, issued on 25 May 2017). Health monitoring did not reveal the presence of any major pathogens (e.g., *Mycobacterium marinum*, *Mycobacterium haemophilum*, *Pseudocapillaria tomentosa*, and *Pseudoloma neurophilia* were below detection threshold) [20]. All fish were wild type ex-breeders due to be euthanized as part of a large migration plan of the fish facility, explaining why so many fish were available and, therefore, used for these experiments.

### 2.2. Chemicals

The anaesthetics used in these experiments are listed in Table 1, with working doses and formulas of all anaesthetic solutions listed in Table 2. Protocols were based on licensed or published anaesthetic or euthanasic doses, with overdose being five to ten times the anaesthetic dose, and author’s experience [9,14,20,21,22,23]. To prevent osmotic shock, all solutions were dissolved in system water. The immersion baths were prepared in 1 L plastic tanks (Tecniplast, Italy) and had a final volume of 0.5 L. Isoeugenol, 2-PE, tricaine and lidocaine HCl were dissolved directly in system water, while clove oil, benzocaine and etomidate were first dissolved using a solvent (96% ethanol (EtOH)) before adding system water. Tricaine and lidocaine HCl solutions were buffered with sodium hydrogen carbonate (NaHCO_3_; Fisher Chemical, UK) at double the anaesthetic concentration (see Table 2). To optimize the anaesthetic dissolution, system water was added with strength in a spraying or fast pouring motion to create a maximum of turmoil. For every replicate tank, the baths were prepared individually and not from stock. Separate tanks with solvent and buffer controls were not included in these experiments since they would not have induced euthanasia and were, therefore, not ethically acceptable for the prospect of this experiment that was designed to compare treatment efficacy. To ease comparison, whenever possible, euthanasic formulations were made with similar concentrations of compounds, buffer, and solvent.

### 2.3. Experiments (Exposure to Anaesthetics)

Four different experiments (see below) were performed all along the hours of a normal working day [10]. The experiments were designed to replicate routine husbandry practices where fish are netted out of a housing tank and immersed into another tank containing the euthanasia solution. In gill breathers, drug administration by bath immersion is equivalent to inhaled drug administration in lung breathers [13,14]. In laboratories, the immersion bath is often not replaced after each euthanasia procedure but reused throughout the day. Similarly, within each experiment (1–4), the drug solution in each euthanasic tank remained unchanged between individual fish/batches. After 10 min of bath immersion, the fish were removed from the euthanasic tanks, counted, and immersed in 2-PE (3 mL/L) overnight before maceration.

#### 2.3.1. Reflexes and Stages of Anaesthesia

For experiments 1 and 2, time to loss of reflexes, i.e., righting reflex, startle reflex, and opercular movements, were recorded. Loss of reflexes such as the righting and startle reflexes is considered external signs of loss of consciousness during anaesthesia (stages III and IV, respectively) [23,24,25]. The righting reflex is the ability to reorient the body when normal upright position is disrupted, and the startle reflex is a Mauthner-initiated rapid muscular response (i.e., a brief twitch) to abrupt stimuli [26]. In the present experimental set-up, the startle reflex was stimulated by tapping at the benchtop surface next to the tank. To avoid startling tanks at the wrong time by propagation of vibrations through surfaces, each tank was set on a different benchtop. For experiments 3 and 4, time to immobility was measured, i.e., the length of time until fish stops swimming after immersion. This is also considered an external sign of loss of consciousness during anaesthesia (stages III and IV) [23,24,25]. Stage V anaesthesia is reached when fish no longer respond to painful stimuli. This stage was not assessed in the present study. Prolonged anaesthetic exposure can lead to medullary collapse (stage VI anaesthesia), with cessation of the opercular beat followed by cardiac arrest [23,24,25], eventually causing death.

#### 2.3.2. Experiment 1—Compound Screening

Experiment 1 assessed time to loss of reflexes after immersion in anaesthetics. Eight tanks containing either isoeugenol, clove oil, 2-PE, buffered tricaine, benzocaine, buffered lidocaine HCl or etomidate were prepared as per formulas in Table 2. The fish were distributed to each tank in batches, one batch at a time, in a non-blinded manner. The number of fish netted per batch was not pre-determined and was limited only by the number of fish in each tank of origin. Each batch size was the result of one netting. The number of batches per treatment group varied in order to reach an even number of fish per treatment. At the end, the total number of fish per treatment was 52–54, divided in 5–16 batches (see Supplementary Table S1 for details). A total of 418 zebrafish aged 18–27 months were included in the experiment. Fish were distributed to treatment at random, with regards to line, age, and sex. The screening tests were performed between August and October 2017.

At 30, 45, 60, 75, 90, 120, 150, 180, 210, 240, 270 and 300 s (seconds) after bath immersion, remaining righting reflexes, opercular beats and startle reflexes of the fish were recorded. Remaining righting reflex was also assessed at 15 s after immersion. For some fish, the startle reflex was lost, then regained after a while, then lost again. Therefore, the recording continued every 60 s after 300 s until 600 s. In the case that fish still displayed reflexes after 10 min, time to loss of reflex was noted as > 600 s.

The results from this experiment (see Section 3.1) indicated that immersion in benzocaine and lidocaine HCl overdoses induced, overall, the most rapid loss of reflexes in adult zebrafish. These anaesthetic agents were, therefore, selected for the additional experiments described below.

#### 2.3.3. Experiment 2—Mass Testing

In the laboratory, there is sometimes a need for larger batches of fish to be euthanized within a short timeframe. The aim of the second experiment was to record time to loss of startle reflexes after mass immersion in baths containing either a benzocaine or a lidocaine HCl overdose. Four tanks, two with benzocaine and two with lidocaine HCl, were used (see formulas in Table 2).

One batch at a time was netted into an immersion bath, and loss of startle reflexes was recorded at 60 s, then every 30 s until 4 min, after immersion. The operator was blind with respect to treatment. The number of fish per batch was not pre-determined and was limited only by the number of fish in each tank of origin, which differed substantially due to the fish in long-term mass spawning set-up being euthanized. Therefore, the number of fish per batch and, hence, the number of batches needed to get an approximately even number of fish per treatment group, varied considerably. Total number of fish per treatment was 304 and 293, divided in 7 and 12 batches, for benzocaine and lidocaine HCl, respectively, (see Supplementary Table S2 for details). Fish were distributed to treatment at random regarding line, age (7–24 months), and sex. The experiment was performed in October 2017.

#### 2.3.4. Experiment 3—Monitoring Discomfort and Aversive Behavior

Several anaesthetic agents are known to induce aversive behavior. The aim of the third experiment was to observe for signs of distress after immersion in baths containing either a benzocaine or a lidocaine HCl overdose (formulas as per Table 2). Four tanks were prepared, two with benzocaine and two with lidocaine HCl. One fish at a time was netted and transferred to a treatment bath, where time to immobility and behavior after immersion were measured. The behavior was scored as either “neutral”, where the fish moved slowly, showing no signs of stress; “husbandry-like”, where the fish displayed behaviors similar to that observed in fish after being transferred to a novel, non-medicated tank, such as moving at fast pace for a short period or exploring the tank; or “aversive”, where the fish appeared very stressed, displaying behaviors not seen in routine husbandry processes, such as erratic swimming, darting, twitching, turning on itself, pushing against the tank wall, rapid opercular movements, and piping or gasping at the surface [8,18,21]. The operator was blind with respect to treatment. For each tank, the experiment was repeated with 28 fish, giving a total of 56 fish per treatment. Altogether, 122 zebrafish of both sexes, aged 7–25 months, were used. The experiment was performed in October 2017.

#### 2.3.5. Experiment 4—Buffering of Lidocaine HCl

In our system water, lidocaine HCl dissolved at 1 g/L gave a pH of 6.8. As the pH of an immersion anaesthetic can affect its efficacy [14,27], and as exposure to acidic solutions may cause irritations and induce aversive behavior in fish [28], lidocaine HCl was buffered prior to fish exposure, with the same concentration ratio of sodium bicarbonate that was used for the tricaine solutions. For experiments 1–3, 1 g/L of lidocaine HCl was buffered with 2 g/L NaHCO_3_, giving a pH of 7.9. In the fourth experiment, different buffers and pH levels were assessed to see if they affected the efficacy of the lidocaine HCl overdose (1 g/L). The buffers tested were 0.2, 2 and 4 mL/L Tris (Sigma-Aldrich, Saint Louis, MI, USA), 1, 2 and 3 g/L NaHCO_3_, and 2 g/L NaHCO_3_ + 50 mL/L 96% EtOH. This concentration of 50 mL/L of 96% EtOH replicates the solvent concentration of the benzocaine treatment. Ten tanks were prepared, one tank for each solution, except for 0.2 and 2 mL/L Tris and 2 g/L NaHCO_3_ + 50 mL/L EtOH that were prepared in duplicates. The pH was measured in each tank prior to fish exposure. One small batch at the time, containing 1–6 fish (see Supplementary Table S3 for batch number and sizes), was netted and transferred to a treatment bath, where time to immobility and behavior after immersion was measured per individual. The behavior was scored as either “neutral”, “husbandry-like” or “aversive”, as for Experiment 3. The operator was blind with respect to treatment. The experiment used 282 zebrafish of both sexes, aged 9–21 months, and was performed in July 2018.

### 2.4. Data Presentation and Statistics

Data were recorded from both individual fish and as batch values. A batch value was determined by the individual fish per batch displaying any reflex the longest. In the main manuscript, the data for individual fish are presented while batch value data can be found in the Supplementary Materials. For each graph, it is indicated if values from individual fish or batches were considered the experimental unit.

For experiment 1, time to loss of startle reflex and opercular movement are presented as Kaplan–Meier curves [29,30]. Time to loss of righting reflexes is presented as stacked bars. The aim of this experiment was to select treatments that lead to prompt disappearance of reflexes for 100% of the fish and not to compare treatment efficacies *per se*. Since each treatment solution was prepared only once and reused for all fish, it can be argued that the experimental unit for this experiment is each anaesthetic tank. Therefore, no assessment of statistical differences between treatments was performed on the data.

For experiment 2, the data are presented as Kaplan–Meier curves and statistical significance between estimates assessed by the log-rank (Mantel–Cox) test [31,32,33]. In experiment 3, difference in time to immobility was assessed by the Wilcoxon rank-sum test [34,35]. Difference in behavior was analysed by the Chi-square test [36] using frequencies. Post hoc tests were performed by analysing 2 × 2 contingency tables (Fisher’s exact test [37]) of the data, with a Bonferroni adjusted significance level (0.05/3 = 0.0167).

To perform statistical analysis for experiment 4, the data from individual groups were pooled into three treatment categories: “Tris” representing 2 and 4 mL/L Tris solutions; “NaHCO_3_” representing 1, 2, and 3 g/L NaHCO_3_ solutions; and “NaHCO_3_ + EtOH” representing 2 g/L NaHCO_3_ + 50 mL/L EtOH 96% solutions (two replicates) (*n* = 98, 126 and 48, respectively). Differences in time to immobility were assessed by the Kruskal–Wallis test [38], followed by Dunn’s post hoc test [39] for multiple comparisons. Difference in behavior was analysed by the Chi-square test using frequencies. Post hoc tests were performed by analysing 2 × 2 contingency tables (Fisher’s exact test) of the data, with a Bonferroni adjusted significance level (0.05/9 = 0.0056). If not otherwise stated, a *p*-value of < 0.05 was considered statistically significant. All statistics were performed, and all figures prepared, using GraphPad Prism 9 (GraphPad Software Inc., San Diego, CA, USA).

## 3. Results

### 3.1. Experiment 1

The anaesthetic overdoses successfully induced deep level anaesthesia with loss of reflexes and opercular beat in all treated fish. However, induction time varied considerably between treatments and endpoints.

**Figure 1:**
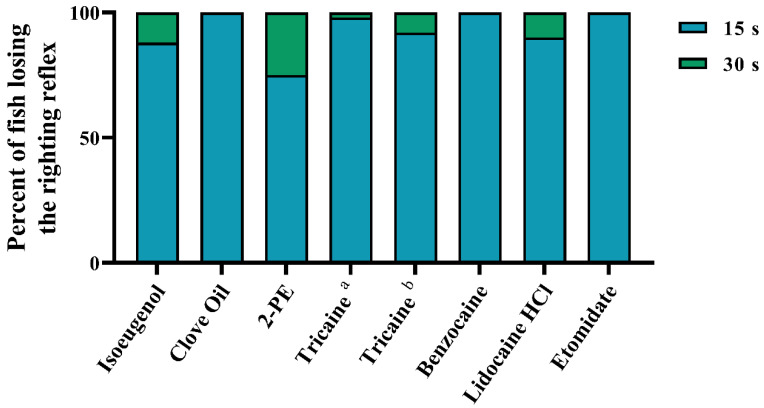
Time to loss of righting reflex after anaesthetic overdose, measured at 15 s intervals after immersion in the experimental solution. Data are presented as percent of total number of fish (*n* = 52–54, depending on treatment, see Supplementary Table S1 for details). ^a^ = 0.5 g/L. ^b^ = 1 g/L.

For loss of the righting reflex, the induction time was similar between the different anaesthetic agents (Figure 1), with all fish losing equilibrium within 30 s after immersion. All fish immersed in clove oil, benzocaine, and etomidate lost equilibrium within 15 s. The least effective treatment was 2-PE, with 25% of the fish maintaining equilibrium after 15 s.

**Figure 2:**
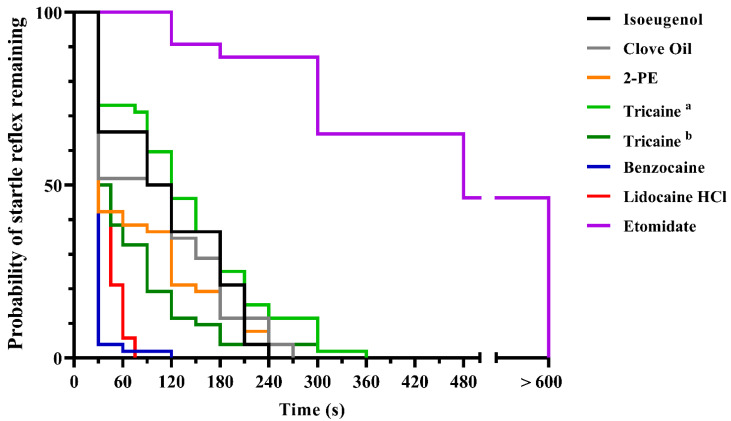
Time to loss of startle reflex in adult zebrafish after induction of anaesthetic overdose, measured at 15 s intervals until 90 s, then every 30 s until 300 s, then every 60 s until 600 s after immersion. Data are presented as a Kaplan–Meier plot describing the probability of fish having intact startle reflex over time (*n* = 52–54, depending on test group, see Supplementary Table S1 for details). ^a^ = 0.5 g/L. ^b^ = 1 g/L.

The differential efficiency of the anaesthetic agents became more apparent when measuring time to loss of the startle reflex (Figure 2). Benzocaine and buffered lidocaine HCl solutions were the most effective, with median induction times of 30 and 37.5 s, and all fish becoming unresponsive within 120 and 75 s, respectively. The least effective compound was etomidate, where close to 50% of the fish remained responsive for over 10 min. For the other compounds, all fish became unresponsive within 240 s after 2-PE and isoeugenol treatments, 270 s after clove oil treatment, and 360 s for both tricaine treatments.

**Figure 3:**
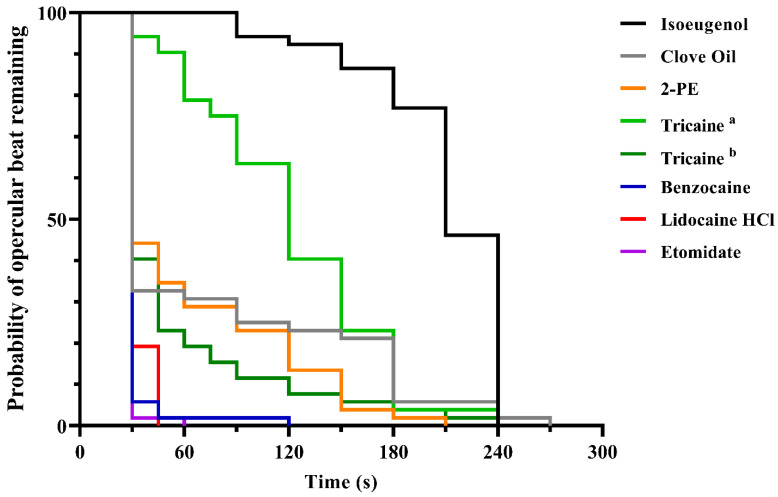
Time to cessation of opercular movements in adult zebrafish after induction of anaesthetic overdose, measured at 15 s intervals until 90 s, then every 30 s until 300 s, after immersion. Data are presented as a Kaplan–Meier plot describing the probability of fish maintaining the opercular beats over time (*n* = 52–54, depending on test group, see Supplementary Table S1 for details). ^a^ = 0.5 g/L. ^b^ = 1 g/L.

Similar discrepancies between the experimental solutions were seen when measuring time to cessation of the opercular beat (Figure 3). The most effective compounds were etomidate, benzocaine, and buffered lidocaine HCl, all with median induction times of 30 s, and with loss of opercular movements achieved for all treated fish within 60, 120 and 45 s, respectively. For 2-PE, tricaine (1 g/L), and clove oil, the results were more variable. While over 50% of the treated fish lost the opercular beat within 30 s, it took 210, 240 and 270 s, respectively, for all fish to lose all movement. Tricaine (0.5 g/L) and isoeugenol were the least effective compounds, with median induction times of 120 s and 210 s, respectively, and opercular beat cessation for the last fish at 240 s for both treatments.

Results based on batch values are presented in Supplementary Figures S1 and S2. A more detailed graphical presentation of the benzocaine and buffered lidocaine HCl data can be found in Supplementary Figures S3 and S4.

### 3.2. Experiment 2

Due to their more efficient performance overall, in the screening in experiment 1, benzocaine and buffered lidocaine HCl were subjected to additional experiments.

**Figure 4:**
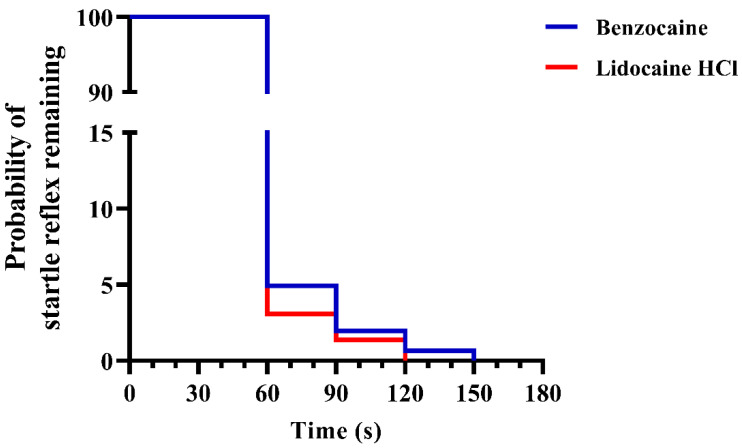
Time to loss of startle reflex in adult zebrafish after a benzocaine or buffered lidocaine HCl overdose induction, when immersed in larger batches (*n* = 5–121 per batch, see Supplementary Table S2 for details). Data are presented as a Kaplan–Meier plot describing the probability of fish having intact startle reflex over time (*n* = 304 and 293, respectively). No statistical differences were detected between treatments (log-rank test).

In experiment 2, larger batches of fish were immersed into the anaesthetic overdoses and time to loss of the startle reflex recorded (Figure 4). After one minute of benzocaine or buffered lidocaine HCl immersion, 95.1% and 96.9% of the fish had become unresponsive, respectively. The induction time for 100% of the fish to have lost responsiveness was 150 and 120 s for benzocaine and buffered lidocaine HCl treatments, respectively. No statistically significant difference was found between the two treatments, as assessed by the log-rank (Mantel–Cox) test. Results based on batch values are presented in Supplementary Figure S5.

### 3.3. Experiment 3

**Figure 5:**
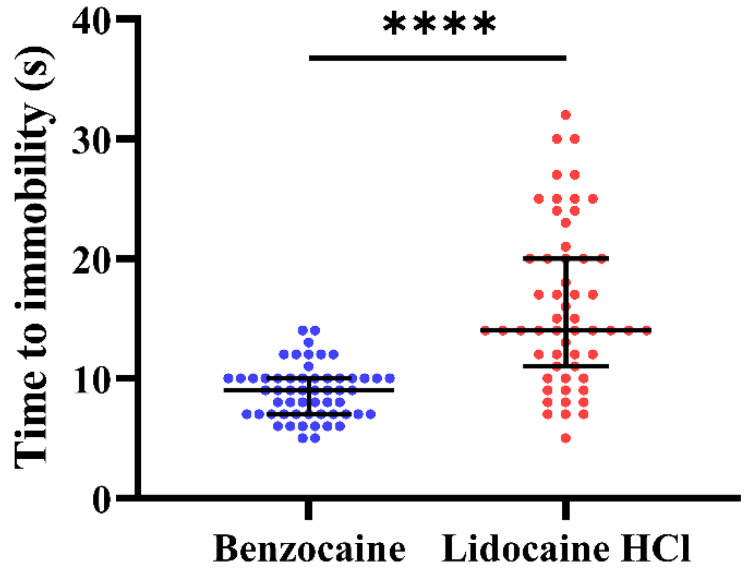
Seconds to immobility after induction to benzocaine or buffered lidocaine HCl overdose (*n* = 56 per treatment). Medians with interquartile ranges are indicated. Asterisks (****) indicate statistical significance (*p* < 0.0001), assessed by the Wilcoxon rank-sum test.

In this experiment, time to immobility and behavior after immersion in benzocaine or buffered lidocaine HCl overdose solutions were recorded. Time to immobility was significantly faster in benzocaine-treated fish compared to buffered lidocaine HCl-treated fish (Wilcoxon rank-sum test; *p* < 0.0001), with median/mean (±SD) induction times of 9/8.8 (±2.2) and 14/16.1 (±6.7) s, respectively (Figure 5).

**Figure 6:**
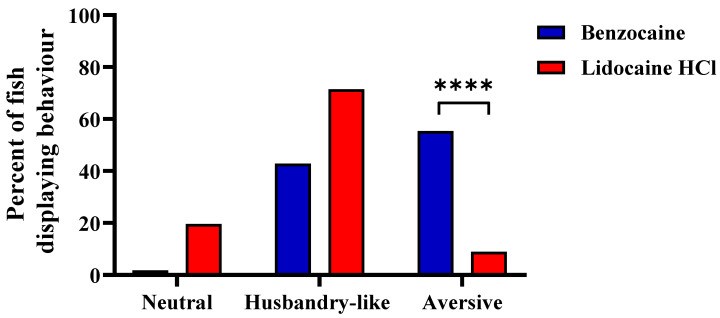
Behavior in adult zebrafish after induction to benzocaine or buffered lidocaine HCl overdose, presented as percentages of categorial outcomes; neutral, husbandry-like, or aversive (see text for description of the behavioral categories) (*n* = 56 per treatment). The behavioral outcome was highly dependent on treatment (Chi-square test; *p* < 0.0001). Post hoc, Fisher’s exact test; ****; *p* < 0.0001.

The fish displayed signs of distress and aversion during exposure to both treatments. However, whether the fish behaved in a neutral, husbandry-like or aversive manner was highly dependent on treatment (Chi-square test, *p* < 0.0001), with the proportion of aversive behavior significantly more pronounced in fish treated with benzocaine than fish treated with buffered lidocaine HCl (post hoc; Fisher’s exact test, *p* < 0.0001) (Figure 6). In benzocaine-treated fish, 55% displayed aversive behavior after immersion, compared to 9% of the buffered lidocaine HCl-treated fish.

### 3.4. Experiment 4

For the final experiment, time to immobility and behavior after immersion in lidocaine HCl overdose baths supplemented with different buffers (inducing different pH levels) were recorded. Time to immobility and pH for each solution are listed in Table 3 and represented in Supplementary Figures S6 and S7. The solution with the lowest pH (7.0) was lidocaine HCl buffered with 0.2 mL/L Tris. Fish treated with this solution reached immobility much slower than the other groups, with a median/mean (±SD) induction time of 63/65.5 (±21.11) s. Furthermore, 20% of these fish showed aversive behavior (see Supplementary Table S4 for details). Testing of 0.2 mL/L Tris was consequently terminated after 10 fish (two batches), and the data omitted from further analysis.

**Figure 7:**
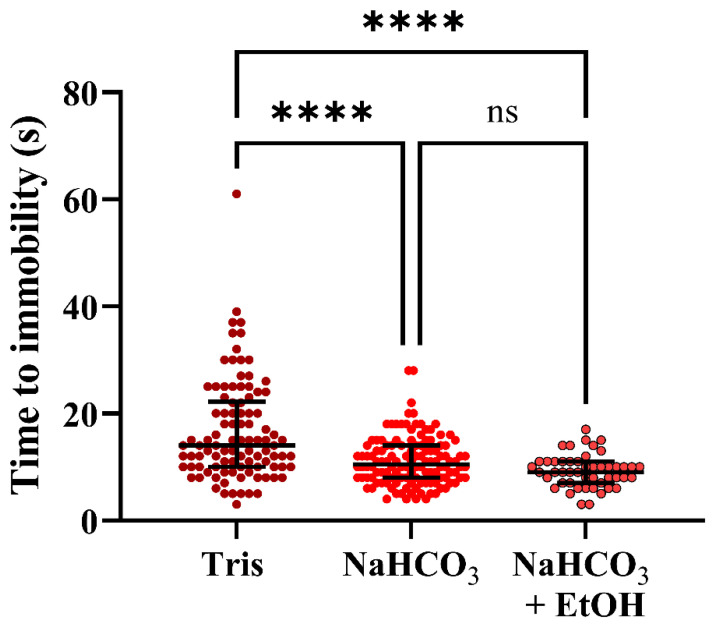
Seconds to immobility in adult zebrafish after immersion in lidocaine HCl (1 g/L) supplemented with various buffers. Data from different subgroups were pooled into three main treatment categories: Tris; 2 and 4 mL/L, NaHCO_3_; 1, 2 and 3 g/L, and 2 g/L NaHCO_3_ + 50 mL/L EtOH (*n* = 98, 126 and 48, respectively, see Supplementary Table S3 for details). Medians with interquartile ranges are indicated. Statistical significance was assessed by the Kruskal–Wallis test, followed by Dunn’s post hoc test for multiple comparisons. ****; *p* < 0.0001. ns = non-significant.

The remaining solutions had more similar pH levels (7.7–8.0) and induction times. The following results were based on the categorical, pooled data, which demonstrated that time to immobility for Tris-treated fish was significantly longer than for NaHCO_3_ and NaHCO_3_ + EtOH -treated fish (Kruskal-Wallis test, followed by Dunn’s post hoc test; *p* < 0.0001), with median/mean (±SD) induction times of 14/16.62 (±9.47) s, 10.5/11.16 (±4.55) s, and 9/9.25 (±3.07) s, respectively (Figure 7).

**Figure 8:**
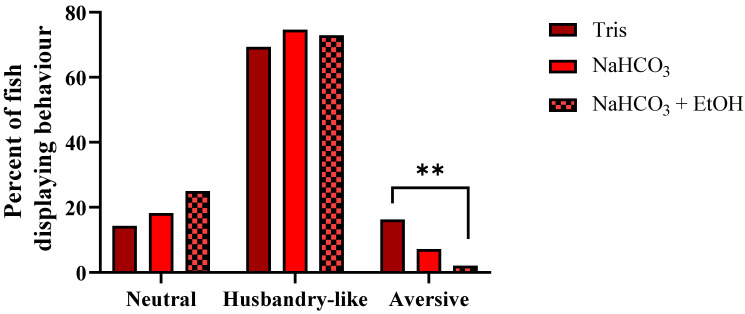
Behavior in adult zebrafish after immersion in lidocaine HCl (1 g/L) supplemented with various buffers. Data from different subgroups were pooled into three main treatment categories: Tris; 2 and 4 mL/L, NaHCO_3_; 1, 2 and 3 g/L, and 2 g/L NaHCO_3_ + 50 mL/L EtOH (*n* = 98, 126 and 48, respectively, see Supplementary Table S3 for details). See text for description of the behavioral categories. The type of behavior was dependent on treatment (Chi-square test; *p* < 0.05). Post hoc, Fisher’s exact test; **; *p* = 0.0063.

Behavioral data for individual treatments are presented in Supplementary Table S4. Regardless of which buffer was used, some fish displayed signs of distress and aversion (Figure 8, Table S4). However, whether the fish behaved in a neutral, husbandry-like or aversive manner depended on the buffer category (pooled data; Chi-square test, *p* < 0.05). The proportion of aversive behavior was significantly more pronounced for fish treated with lidocaine HCl buffered with Tris than those treated with the NaHCO_3_ + EtOH buffered solution (post hoc; Fisher’s exact test, *p* = 0.0063). Of fish immersed in the Tris buffer, 16.3% displayed aversive behavior, compared to 2.1% of those immersed in the NaHCO_3_ + EtOH buffer. For those immersed in NaHCO_3_ buffered lidocaine HCl, 7.14% displayed aversive behavior.

## 4. Discussion

Because zebrafish are one of the most used laboratory animals in the world, efficient, humane, and reliable protocols for their euthanasia are essential. The present study aimed at screening commonly used anaesthetics agents for overdose induction efficacy in adult zebrafish, and subsequently select the most effective formulations inducing the least aversive behavior for this species.

### 4.1. Experiments 1 and 2; Screening and Mass Testing

In experiment 1, overdose induction efficacy was assessed by recording loss of reflexes during exposure. At the concentrations tested here, all fish lost the righting reflex within 30 s of exposure, indicating that stage III anaesthesia [23,24,25] was achieved quickly by all compounds. The results agree with previous studies on zebrafish equilibrium loss following lidocaine HCl, benzocaine, clove oil, etomidate and tricaine immersion [8,9,21,22,23,40,41,42], though the present induction times were generally shorter. This is likely due to the higher doses used in this study.

Time to loss of the startle reflex and the opercular movement was less consistent between the different anaesthetics. The most effective treatment was buffered lidocaine HCl. Within 45 s, all fish had lost the opercular beat, and within 75 s, all fish had become unresponsive. This is slightly faster than what has previously been reported from zebrafish [9,21], but not surprising as our dose was higher (0.35–0.6 g/L vs. 1 g/L). Benzocaine was the second most effective treatment, with all fish losing the startle reflex and opercular movements within 120 s. Although the benzocaine induction time was generally short, and with lower median scores than lidocaine HCl, the results were somewhat less consistent than those from lidocaine HCl immersion, as some fish kept intact reflexes up to four times as long as the treatment average. Previous studies following adult zebrafish during benzocaine treatment report longer induction times to reach deep anaesthesia than here, but this is likely explained by dose differences (0.1 g/L and 200 ppm vs. 1 g/L) [23,43].

While all fish immersed in lidocaine HCl and benzocaine solutions had lost reactivity within 2 min, the other experimental compounds were less reliable, showing longer and more variable induction times. During clove oil, 2-PE and tricaine (1 g/L) treatments, about half the fish lost the startle reflex and the opercular beat within 30 s. For all fish to become unresponsive, however, an additional 210–330 s of exposure was needed, which indicate large individual variation of the anaesthetic effects. Similar patterns, although less potent, was seen during isoeugenol and tricaine (0.5 g/L) immersion. A possible explanation to this might lie in our experimental design. As one experimental solution per treatment was reused through the screening, there is a risk that the potency of the anaesthetic agent was reduced after several rounds of exposure, thus increasing the induction time of the last batches, and causing the variability in the data. When comparing average induction time between the first half of fish with the last half (see supplementary Table S5), an increase was seen for the later batches of 2-PE and tricaine (both doses) -treated fish. This was particularly apparent during 0.5 g/L tricaine treatment, where later batches had a 77 s slower average than early batches. Conversely, for clove oil-treated fish, this relationship was inverse, with the first half of fish having longer induction time than the last half. No difference was found between early and late batches of benzocaine, buffered lidocaine HCl or etomidate exposed fish. Moreover, individual fish responding outside the general pattern were observed in both early and late batches for most treatments. Surprisingly, there was not necessarily a correlation between time to loss of startle reflex and time to loss of opercular beat. For instance, for isoeugenol-treated fish, the variation in time to loss of the startle reflex generally came from early batches, while the variation in time to loss of the opercular beat generally came from late batches, meaning that these observations were from different fish. In sum, some of the anaesthetic solutions may have lost or gained potency from the first to the last batch, but not to such an extent that the general conclusion is affected: benzocaine and buffered lidocaine HCl were the most efficient treatments.

Clove oil is an essential oil extracted from *Syzygium aromaticum*, containing eugenol as the main active ingredient (70–95%) [22]. The induction time described above, including its large variation, agrees with a previous report using clove oil overdose for adult zebrafish euthanasia [44]. Isoeugenol is another active component of clove oil, although to a much lesser extent than eugenol. Here, we used Aqui-S, that contains 540 mg/mL isoeugenol. While a survival rate of < 30% was seen after 150 ppm Aqui-S exposure for 15 min [43], there is, to our knowledge, no studies describing overdose induction efficacy for isoeugenol or Aqui-S in zebrafish. Still, times to reach stage V anaesthesia that have been reported are comparable to the current results, though somewhat slower, again probably due to lower doses [45]. Although 2-PE is used in laboratories for zebrafish anaesthesia and euthanasia [7,46], there are, to our knowledge, no published scientific studies assessing its overdose induction efficacy specifically for adult zebrafish.

Tricaine efficacy, on the other hand, is well studied in zebrafish [8,9,10,22,44,47,48,49]. However, reports are conflicting regarding stage VI induction time. While Wilson et al. [8] found that unbuffered 0.25 g/L tricaine induced opercular beat cessation in about 50 s, Collymore and colleagues [9] reported an average of 500 s to loss of opercular beat during 0.25 g/L buffered tricaine treatment. It would appear then that buffering the tricaine solution could increase the induction time, but this is contradicted by a report from Davis et al. [44] where 1.7 mM (approx. 0.44 g/L) unbuffered tricaine induced stage VI in just under 3 min. The latter result is more similar to the median time of 120 s for 0.5 g/L buffered tricaine observed presently. Combining the variability in induction with the many reports of aversive behavior seen during treatment in zebrafish, makes tricaine a poor choice for anaesthesia overdose in this species. Indeed, compared with benzocaine and buffered lidocaine HCl, the time to loss of reflexes with tricaine-induced overdose was less consistent along the day, suggesting that tricaine baths should be changed after a few fish. Whatever protocol was used here, the absence of righting reflex revealed that fish were anaesthetized while startle reflex and opercular beat remained. Still, the prompt loss of reflexes with benzocaine and buffered lidocaine HCl allows a faster completion of euthanasia. Thus, without compromising animal welfare, fresher samples can be obtained with these anaesthetics than with tricaine. Moreover, tricaine-induced aversive behaviors have been reported in a high percentage of fish (30–90%) [8,9], whereas the present protocols for buffered lidocaine HCl triggered aversive behaviors in 2–10% of fish. Overall, buffered lidocaine HCl seems a refined alternative to tricaine for the induction of euthanasia by overdose in adult zebrafish.

The least effective treatment from experiment 1 was etomidate. Etomidate is a non-barbiturate hypnotic agent that can induce zebrafish anaesthesia without analgesia [50,51]. It has been suggested as an alternative to tricaine for zebrafish anaesthesia and euthanasia, as it induces less aversive behavioral responses [12,25]. Here, etomidate treatment led to rapid loss of righting reflex and opercular beat in all fish, after 15 s and 60 s, respectively, which indicated that this agent was an effective anaesthetic in zebrafish. However, nearly 50% of the etomidate-treated fish maintained an intact startle response for an additional 9 (or more) minutes. It is possible that these fish would recover if transferred to non-medicated water, which suggests that etomidate is not a good candidate for zebrafish euthanasia, at least not within this time frame. Indeed, an intact startle response after 10 min immersion in a very high dose (50 mg/L) suggests that etomidate does not even induce surgical stage anaesthesia and should not be used for invasive procedures in this species. This is supported by other zebrafish studies [40,51], although at much lower concentrations (0.2–2 mg/L). The results from the etomidate screening here stresses the need to check for multiple signs of death during anaesthetic overdose, as observation of opercular movement termination alone does not suffice. They also reinforce that fish should be left in the overdose solution at least 10 min after opercular beat cessation, and that death should be confirmed by physical means such as maceration (i.e., two stage euthanasia).

**Figure 9:**
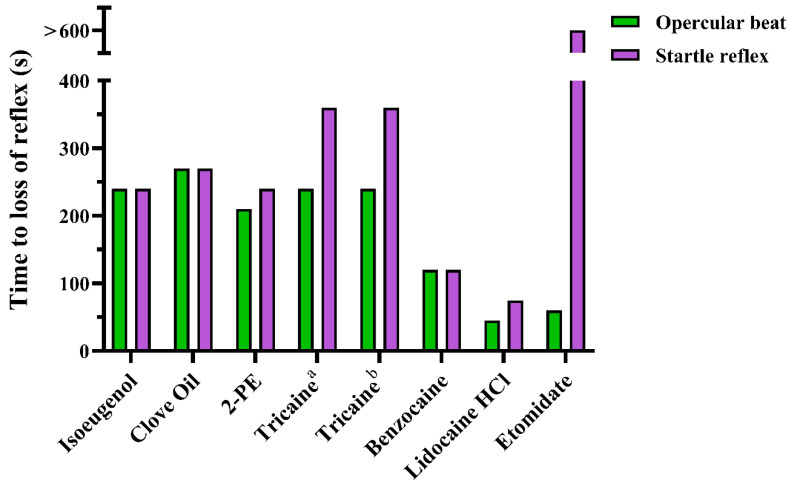
Comparison of time to loss of opercular beat and startle reflex per treatment in experiment 1. The results are based on the time at which 100 % of fish had lost the reflexes. ^a^ = 0.5 g/L. ^b^ = 1 g/L.

According to literature describing the levels of anaesthesia, loss of responsiveness occurs prior to cessation of opercular movement [24,25,52,53]. Conversely, during all anaesthetic treatments tested here, fish remained responsive up until or after respiration stopped (Figure 9). It might be that the stages of euthanasia, induced rapidly with an overdose, do not precisely follow the stages of anaesthesia, induced more slowly with lower doses. This highlights that external signs of loss of consciousness must be interpreted with care, and that absence of opercular beat can be a misleading clinical sign when assessing fish death. This complexity is also demonstrated by the early but temporary disappearance of the startle reflex in some fish. This can be a challenge for the experimental design if time points to detect absence of a reflex are set too early. In these experiments, fish were observed for more than five minutes after opercular beats stopped, and it is very likely that a late recovery of the startle reflex would have been detected. As an ethical decision, we chose not to assess fish recovery following immersion in an overdose of anaesthetic. However, we do not know whether there is correlation between the presence or final disappearance of the startle reflex and the ability of the fish to recover consciousness, i.e., whether the fish is alive or dead, since neuromuscular reactions may remain for a few minutes after death. The startle reflex was our most valuable indicator since it was the one lasting the longest after induction (Figure 9), but we cannot presume that its presence indicates that fish are still alive, and that its final disappearance reveals a time of death. This highlights the importance of completing euthanasia by a second procedure to ensure permanent cessation of brain activity and blood circulation.

Based on the results from experiment 1, benzocaine and buffered lidocaine HCl were submitted to additional testing. First off was the mass testing (experiment 2), where larger batches of fish were euthanized. For this experiment, only time to loss of startle reflex was measured, as this was the reflex that remained intact the longest in experiment 1 and the only reflex observable in large batches. Times to loss of startle reflex observed during mass testing were comparable to those in the initial screening. Within one minute, over 95% of the fish had become unresponsive. As in experiment 1, some fish remained responsive for longer, but within 2 min all buffered lidocaine HCl treated fish had lost responsiveness, and after 2 min 30 s the benzocaine-treated fish had done the same. Both treatments therefore seem reliable and effective, and work well with repetitive use and large batches.

### 4.2. Experiments 3 and 4; Behavior and Buffers

Next, as many anaesthetic agents can be aversive, we assessed behavioral responses during benzocaine and buffered lidocaine HCl immersion (experiment 3). At the doses tested here, benzocaine treatment induced significantly more distress than lidocaine, with over half the benzocaine treated fish displaying adverse behavior. In previous reports from zebrafish, results are conflicting. For instance, no erratic behavior was seen during 200 ppm benzocaine treatment [43], while 50 mg/L benzocaine-induced avoidance related behavior in a preference test [12]. In other species, benzocaine immersion, even at low doses, induced stress responses or aversive/avoidance behavior; medaka (*Oryzias latipes*) (50 mg/L), Atlantic salmon (*Salmo salar*) (40 mg/L), Atlantic cod (*Gadus morhua*) (20 mg/L), Atlantic halibut (*Hippoglossus hippoglossus*) (40 mg/L), and bony bream (*Nematalosa erebi*) (100 mg/L) [15,54,55]. On the other hand, no aversion was seen in fathead minnow (*Pimephales promelas*) (50 mg/L), carp (*Cyprinus carpio*) (50 mg/L), rainbow trout (*Oncorhynchus mykiss*) (50 and 150 mg/L) during benzocaine treatment [15,56]. These results clearly indicate strong species specificity for benzocaine aversiveness. However, the above-mentioned studies investigated relatively low doses compared to the present study, where fish were immerged in 1 g/L benzocaine (10 × the recommended dose for anaesthesia (100 mg/L)). So, it is possible that the involved species would respond differently to an overdose concentration. While benzocaine treatment was more aversive than buffered lidocaine HCl treatment, still, 9% of lidocaine treated fish displayed erratic behavior. Here too, results from other zebrafish studies are conflicting. For instance, 1 out of 10 fish treated with either 0.4 g/L or 0.5 g/L lidocaine HCl displayed erratic behavior [9], which agrees with our findings. But no distress was seen in zebrafish treated with either 0.3–0.35 g/L or 0.6 g/L lidocaine HCl [9,21]. In contrast, 50 mg/L lidocaine HCl was reported aversive for zebrafish in a preference test [12]. It is unknown to us what might cause the inconsistency between dose and aversiveness for lidocaine in zebrafish. However, in the latter case, the lidocaine HCl solution was unbuffered, which, although the concentration was low, might have contributed to the fish not preferring the solution. In summary, benzocaine leads to fast immobilization, and rapid loss of reflexes, as seen in experiments 1 and 2, but the high occurrence of aversive behavior during immersion gives cause for concern using this agent for zebrafish overdose induction. Conversely, buffered lidocaine HCl treatment has slightly slower induction time to immobility but leads to very rapid loss of reflexes and relatively low aversiveness. We, therefore, recommend buffered lidocaine HCl as the most suitable anaesthetic in adult zebrafish for the induction of euthanasia by overdose.

In experiment 4, we wanted to see if we could optimize the lidocaine HCl protocol to be even more efficient and less aversive by altering buffers and pH. The results demonstrated that not only pH, but also the choice of buffer, affected time to immobility and behavior during exposure. It early became clear that lidocaine HCl buffered to pH 7.0 using Tris led to long induction time and many cases of aversive behavior, so this treatment was quickly terminated (after two batches/ten fish). We did not test if lidocaine HCl buffered to the same pH with NaHCO_3_ or NaHCO_3_ + EtOH would give similar results. However, the pH levels of the other lidocaine HCl with buffer solutions tested were comparable, and here, Tris still performed worse than NaHCO_3_ or NaHCO_3_ + EtOH buffers, both in terms of induction time and in terms of causing distress during immersion. Why NaHCO_3_ was more effective than Tris is not known to us, but it might be that the CO_2_ (released when NaHCO_3_ reacts with water) contributes to overdose induction [14]. A general observation for both Tris- and NaHCO_3_-buffered solutions was that increased pH correlated with shorter induction time. This has also previously been demonstrated with lidocaine [27] and etomidate [50], but is not necessarily true for all anaesthetic agents, for instance, as discussed above regarding tricaine. However, this is particularly relevant for a molecule such as lidocaine with a pKa of 7.75 (see Table 2 for pKa of active chemicals). When pH is below pKa, lidocaine is mainly present in solution as an ionized molecule that is poorly absorbed through the gills. When pH reaches above pKa, the non-ionized form is dominant, and the gills can consequently absorb much more lidocaine. This potentially explains the difference in efficacy between pH 7.0 and pH > 7.7 [40].

The most effective buffer was NaHCO_3_ + EtOH, having both the shortest induction time and causing the least distress. This suggests that EtOH either increases the efficacy or uptake of lidocaine and/or contributes to overdose induction and behavior alterations on its own. Regarding the former, it is possible that ethanol increases the apparent solubility of the non-ionized lidocaine form and, therefore, may increase the amount of lidocaine absorbed through the gills [57]. For the latter, here we added 50 mL EtOH per L solution, giving an EtOH concentration of 5%. Previous reports imply that 3% EtOH cause erratic behavior in zebrafish, but daily exposure of 1 h for two weeks did not cause mortality [58,59]. On the other hand, Mathur et al. [60] found that 1.5% EtOH was attractive to zebrafish during a preference test, while 20 min exposure to 2.5 and 3.5% EtOH was deadly to the fish. They also noted erratic movements after 10 min immersion at the highest concentrations. In experiments 1–3, 5% EtOH was also added as a solvent to the benzocaine solutions. Therefore, it is possible that the aversive behavior seen after benzocaine immersion was affected and/or caused by the high EtOH content, yet that would be the opposite effect to what we saw in experiment 4 with lidocaine. For future studies, lower solvent concentrations should be assessed. However, as EtOH can induce erratic behavior but hardly any aversion or erratic behavior was seen here during lidocaine HCl + NaHCO_3_ + EtOH immersion, there is likely a positive interaction from either two or all three elements that caused this outcome. Of note, for fish treated with lidocaine HCl + 2 g/L NaHCO_3_, the percentage of fish demonstrating aversive behavior was almost identical between experiments 3 and 4. This is interesting because, in experiment 3, fish were anesthetized individually, while, in experiment 4, fish were anesthetized in batches. As zebrafish normally swim in shoals, isolation would likely be a stressor, however, no additional distress was seen when fish were treated individually. In summary, 1 g/L lidocaine HCl buffered with 2 g/L NaHCO_3_ + 50 mL/L EtOH proved to decrease both induction time and aversive behavior, and further improve euthanasia induction.

## 5. Conclusions

Welfare of animals during their stay in the laboratories, as well as during euthanasia, is a topic that requires attention for all fish caretakers and researchers. It is likely that the extensive use of zebrafish as a research model will only continue to grow in the future; therefore, a safe, humane, and effective method of euthanasia for this species is necessary. In the present study, screening of commonly used anaesthetic agents for overdose euthanasia revealed 1 g/L lidocaine hydrochloride buffered with 2 g/L NaHCO_3_ could induce loss of all reflexes within 2 min, be reliable and provoke little aversive behavior in adult zebrafish. By adding an additional 50 mL/L EtOH to the lidocaine HCl with NaHCO_3_ solution, the induction time, and aversive behavior, were reduced even further. While having high efficacy, 1 g/L benzocaine was too aversive to be recommended for zebrafish euthanasia. It should be noted, though, that for both lidocaine HCl and benzocaine treatments, only one concentration was tested, and that it is possible that lower doses could have the same efficacy but cause less aversion. It is also possible that some anaesthetic agents tested here, although slower at inducing euthanasia, would cause a more peaceful death, with even less signs of aversion than buffered lidocaine HCl. This should be topics for future studies. Finally, hypothermic shock has been suggested a suitable alternative to anaesthetic overdose in zebrafish. This is based on both the rapid induction time using ice water immersion and the aversiveness of some anaesthetics (e.g., tricaine) [18]. However, as supported by the data presented here, the issues with some specific anaesthetics should not be interpreted as a generality for all anaesthetic overdose protocols. Although it is likely that hypothermic shock induces a faster loss of observable reflexes, we do not know whether this is due to thermic inhibition of muscles or a loss of consciousness. Hence, we cannot presume fish experience during hypothermic shock [7]. Moreover, fish display aversive reaction at the start of hypothermic immersion, sometimes in much higher percentages (39%) [8] than what was observed with buffered lidocaine HCl (<10%). Therefore, it is possible that hypothermic shock induces a faster but more aversive death than buffered lidocaine HCl overdose. The two protocols should be compared further using behavioral, physiological, and biochemical indicators of fish welfare and experience. This comparison would be particularly interesting to perform for all developmental stages of zebrafish. Indeed, hypothermic shock is not as efficient in early stages as in adult fish [19]. Data presented here only focus on adult *Danio rerio*, and experiments with lidocaine should be performed on embryos and larvae to assess the anaesthetic’s ability to induce euthanasia in all developmental stages. Based on the current study, we recommend 1 g/L lidocaine HCl buffered with 2 g/L NaHCO_3_ and 50 mL/L 96% ethanol as a humane, reliable, and effective method for induction of euthanasia by overdose in adult zebrafish.

## Figures and Tables

**Table 1:** Overview of experimental anaesthetics.

Generic Name	Active Chemical	Product Name	Manufacturer
Isoeugenol	2-Methoxy-4-(prop-1-en-1-yl)phenol	AQUI-S VET (540 mg/mL)	SCAN AQUA (Norway)
Clove Oil	2-Methoxy-4-(prop-2-en-1-yl)phenol (i.e., eugenol) and others unidentified	Clove Oil	Boots (UK)
2-PE	2-Phenoxyethanol	Aqua-Sed	VETARK (UK)
Tricaine	Ethyl 3-aminobenzoate methanesulfonate	Tricaine Pharmaq 1000 mg/g	PHARMAQ (UK)
Benzocaine	Ethyl 4-aminobenzoate	Benzocaine (06952)	SIGMA-ALDRICH (USA)
Lidocaine HCl	2-(diethylamino)-N-(2,6-dimethylphenyl)acetamide hydrochloride monohydrate	Lidocaine hydrochloride monohydrate (L5647)	SIGMA-ALDRICH (USA)
Etomidate	(R)-1-(α-Methylbenzyl)imidazole-5-carboxylic acid ethyl ester	Etomidate CRS (E2503000)	European Pharmacopoeia (EP) Reference Standard

**Table 2:** Formulas for anaesthetic overdoses in experiments 1 to 3 and pKa of anaesthetics. Clove oil contains eugenol and other potentially active compounds (e.g., terpinoids). Only molecules known to affect the pH of the anaesthetic solutions are buffered (i.e., tricaine and lidocaine HCl). Compounds lacking solubility at the sought concentrations are first dissolved in ethanol. SW = system water. N/A = not applicable. ^a^ = 0.5 g/L. ^b^ = 1 g/L.

Agent	Dose	pKa
Isoeugenol	540 mg/L SW	10.01
Clove Oil	0.1% (1:10 in EtOH, then 10 mL/L SW)	N/A
2-PE	3 mL/L SW	15.1
Tricaine ^a^	0.5 g/L + 1 g/L NaHCO_3_ SW	2.89
Tricaine ^b^	1 g/L + 2 g/L NaHCO_3_ SW	2.89
Benzocaine	1 g/L (20 g/L in EtOH, then 50 mL/L SW)	2.78
Lidocaine HCl	1 g/L + 2 g/L NaHCO_3_ SW	7.75
Etomidate	50 mg/L (10 mg/mL in EtOH, then 5 mL/L SW)	4.25

**Table 3:** pH vs. Time to immobility for lidocaine HCl (1 g/L) with different buffers, experiment 4.

Treatment	pH	Seconds to Immobility		*n*
		Mean (±SD)	Median	
Tris (0.2 mL/L)	7.0	65.5 (±21.11)	63	10
Tris (2 mL/L)	7.9	20 (±10.12)	18	57
Tris (4 mL/L)	8.0	11.93 (±5.93)	10	41
NaHCO_3_ (1 g/L)	7.7	12.5 (±4.91)	12	38
NaHCO_3_ (2 g/L)	7.9	10.76 (±4.54)	10	51
NaHCO_3_ (3 g/L)	8.0	10.32 (±3.97)	10	37
**NaHCO_3_ (2 g/L) + 50 mL/L EtOH**	8.0	9.25 (±3.07)	9	48

## Data Availability

Original data are available on request.

## Supplementary Materials

The following are available online at https://www.mdpi.com/article/10.3390/biology10111133/s1, Figure S1: Probability of remaining startle reflex after immersion in experimental solution, batch results, Figure S2: Probability of remaining opercular beat after immersion in experimental solution, batch results, Figure S3: Probability of remaining startle reflex; details benzocaine vs. lidocaine HCl, Figure S4: Probability of remaining opercular beat; details benzocaine vs. lidocaine HCl, Figure S5: Probability of remaining startle reflex after exposure to benzocaine or lidocaine in mass testing, batch results, Figure S6: Time to immobility in after immersion in lidocaine HCl supplemented with various buffers, Figure S7: Time to immobility in after immersion in lidocaine HCl supplemented with various buffers, batch results, Table S1: Number of fish per batch (Experiment 1), Table S2: Number of fish per batch (Experiment 2), Table S3: Number of fish per batch (Experiment 4), Table S4: Behavior in fish after exposure to 1g /L lidocaine HCl supplied with different buffers (Experiment 4), Table S5: Average time to loss of reflexes in first vs. second half of experimental subjects (Experiment 1).

## References

[1] European Union (2010). Directive 2010/63/EU of the European Parliament and of the Council; On the protection of animals used for scientific purposes. Off. J. Eur. Union.

[2] Staib-Lasarzik I., Kriege O., Timaru-Kast R., Pieter D., Werner C., Engelhard K., Thal S.C. (2014). Anesthesia for Euthanasia Influences mRNA Expression in Healthy Mice and after Traumatic Brain Injury. J. Neurotrauma.

[3] Gause B.R., Trushenski J.T., Bowzer J.C., Bowker J.D. (2012). Efficacy and Physiological Responses of Grass Carp to Different Sedation Techniques: I. Effects of Various Chemicals on Sedation and Blood Chemistry. N. Am. J. Aquac..

[4] Matsche M.A. (2017). Efficacy and Physiological Response to Chemical Anesthesia in Wild Hickory Shad during Spawning Season. Mar. Coast. Fish..

[5] Kinth P., Mahesh G., Panwar Y. (2013). Mapping of Zebrafish Research: A Global Outlook. Zebrafish.

[6] European Commission (2020). 2019 Report on the Statistics on the Use of Animals for Scientific Purposes in the Member States of the European Union in 2015–2017.

[7] Lidster K., Readman G.D., Prescott M.J., Owen S. (2017). International survey on the use and welfare of zebrafish Danio rerio in research. J. Fish Biol..

[8] Wilson J.M., Bunte R.M., Carty A.J. (2009). Evaluation of Rapid Cooling and Tricaine Methanesulfonate (MS222) as Methods of Euthanasia in Zebrafish (Danio rerio). J. Am. Assoc. Lab. Anim. Sci..

[9] Collymore C., Banks E.K., Turner P.V. (2016). Lidocaine Hydrochloride Compared with MS222 for the Euthanasia of Zebrafish (*Danio rerio*). J. Am. Assoc. Lab. Anim. Sci..

[10] Sánchez-Vázquez F.J., Terry M.I., Felizardo V.O., Vera L.M. (2011). Daily Rhythms of Toxicity and Effectiveness of Anesthetics (MS222 and Eugenol) in Zebrafish (Danio Rerio). Chronobiol. Int..

[11] Wong D., Von Keyserlingk M.A.G., Richards J.G., Weary D.M. (2014). Conditioned Place Avoidance of Zebrafish (*Danio rerio*) to Three Chemicals Used for Euthanasia and Anaesthesia. PLoS ONE.

[12] Readman G.D., Owen S., Murrell J.C., Knowles T. (2013). Do Fish Perceive Anaesthetics as Aversive?. PLoS ONE.

[13] Schroeder P., Lloyd R., McKimm R., Metselaar M., Navarro J., O’Farrell M., Readman G.D., Speilberg L., Mocho J.-P. (2021). Anaesthesia of laboratory, aquaculture and ornamental fish: Proceedings of the first LASA-FVS Symposium. Lab. Anim..

[14] Neiffer D.L., Stamper M.A. (2009). Fish Sedation, Anesthesia, Analgesia, and Euthanasia: Considerations, Methods, and Types of Drugs. ILAR J..

[15] Readman G.D., Owen S., Knowles T., Murrell J.C. (2017). Species specific anaesthetics for fish anaesthesia and euthanasia. Sci. Rep..

[16] Park I.-S., Gil H.W., Lee T.H., Nam Y.K., Lim S.G., Kim D.S. (2017). Effects of Clove Oil and Lidocaine-HCl Anesthesia on Water Parameter during Simulated Transportation in the Marine Medaka, Oryzias dancena. Dev. Reprod..

[17] Strykowski J.L., Schech J.M. (2015). Effectiveness of Recommended Euthanasia Methods in Larval Zebrafish (*Danio rerio*). J. Am. Assoc. Lab. Anim. Sci..

[18] Matthews M., Varga Z.M. (2012). Anesthesia and Euthanasia in Zebrafish. ILAR J..

[19] Wallace C.K., Bright L.A., Marx J.O., Andersen R.P., Mullins M.C., Carty A.J. (2018). Effectiveness of Rapid Cooling as a Method of Euthanasia for Young Zebrafish (*Danio rerio*). J. Am. Assoc. Lab. Anim. Sci..

[20] Mocho J.-P., Martin D.J., Millington M.E., Torres Y.S. (2017). Environmental Screening of Aeromonas hydrophila, *Mycobacterium* spp., and Pseudocapillaria tomentosa in Zebrafish Systems. J. Vis. Exp..

[21] Collymore C., Tolwani A., Lieggi C., Rasmussen S. (2014). Efficacy and Safety of 5 Anesthetics in Adult Zebrafish (*Danio rerio*). J. Am. Assoc. Lab. Anim. Sci..

[22] Grush J., Noakes D.L.G., Moccia R.D. (2004). The Efficacy of Clove Oil As An Anesthetic for the Zebrafish, *Danio rerio* (Hamilton). Zebrafish.

[23] Olt J., Allen C.E., Marcotti W. (2016). In vivo physiological recording from the lateral line of juvenile zebrafish. J. Physiol..

[24] McFarland W.N., Klontz G.W. (1969). Anesthesia in fishes. Fed. Proc..

[25] Close B., Banister K., Baumans V., Bernoth E.-M., Bromage N., Bunyan J., Erhardt W., Flecknell P., Gregory N., Hackbarth H. (1997). Recommendations for euthanasia of experimental animals: Part 2. Lab. Anim..

[26] Machnik P., Schirmer E., Glück L., Schuster S. (2018). Recordings in an integrating central neuron provide a quick way for identifying appropriate anaesthetic use in fish. Sci. Rep..

[27] Carrasco S., Sumano H., Navarro-Fierro R. (1984). The use of lidocaine-sodium bicarbonate as anaesthetic in fish. Aquaculture.

[28] Sneddon L.U. (2019). Evolution of nociception and pain: Evidence from fish models. Philos. Trans. R. Soc. B Biol. Sci..

[29] Kaplan E.L., Meier P. (1958). Nonparametric Estimation from Incomplete Observations. J. Am. Stat. Assoc..

[30] Rich J.T., Neely J.G., Paniello R.C., Voelker C.C.J., Nussenbaum B., Wang E. (2010). A practical guide to understanding Kaplan-Meier curves. Otolaryngol. Neck Surg..

[31] Mantel N. (1966). Evaluation of survival data and two new rank order statistics arising in its consideration. Cancer Chemother. Rep..

[32] Peto R., Peto J. (1972). Asymptotically Efficient Rank Invariant Test Procedures. J. R. Stat. Soc. Ser. A.

[33] Harrington D., Armitage P., Colton T. (2005). Linear Rank Tests in Survival Analysis. Encyclopedia of Biostatistics.

[34] Wilcoxon F. (1945). Individual Comparisons by Ranking Methods. Biom. Bull..

[35] Mann H.B., Whitney D.R. (1947). On a Test of Whether one of Two Random Variables is Stochastically Larger than the Other. Ann. Math. Stat..

[36] McHugh M.L. (2013). The Chi-square test of independence. Biochem. Med..

[37] Agresti A. (1992). A Survey of Exact Inference for Contingency Tables: Rejoinder. Stat. Sci..

[38] Kruskal W.H., Wallis W.A. (1952). Use of Ranks in One-Criterion Variance Analysis. J. Am. Stat. Assoc..

[39] Dunn O.J. (1961). Multiple Comparisons among Means. J. Am. Stat. Assoc..

[40] Jorge S., Ferreira J.M., Olsson I.A.S., Valentim A.M. (2021). Adult Zebrafish Anesthesia: A Study of Efficacy and Behavioral Recovery of Different Anesthetics. Zebrafish.

[41] Valentim A., Félix L., Carvalho L., Diniz E., Antunes L. (2016). A New Anaesthetic Protocol for Adult Zebrafish (*Danio rerio*): Propofol Combined with Lidocaine. PLoS ONE.

[42] Nordgreen J., Tahamtani F.M., Janczak A.M., Horsberg T.E. (2014). Behavioural Effects of the Commonly Used Fish Anaesthetic Tricaine Methanesulfonate (MS-222) on Zebrafish (Danio rerio) and Its Relevance for the Acetic Acid Pain Test. PLoS ONE.

[43] Ehrlich O., Karamalakis A., Krylov A.J., Dudczig S., Hassell K.L., Jusuf P.R. (2019). Clove Oil and AQUI-S Efficacy for Zebrafish Embryo, Larva, and Adult Anesthesia. Zebrafish.

[44] Davis D.J., Klug J., Hankins M., Doerr H.M., Monticelli S.R., Song A., Gillespie C.H., Bryda E.C. (2015). Effects of Clove Oil as a Euthanasia Agent on Blood Collection Efficiency and Serum Cortisol Levels in Danio rerio. J. Am. Assoc. Lab. Anim. Sci..

[45] Musk G.C., Ezzy B.J., Kenchington L.M., Hopper W.A., Callahan L.M. (2020). A Comparison of Buffered Tricaine Methanesulfonate (MS-222) and Isoeugenol Anesthesia for Caudal Fin Clipping in Zebrafish (*Danio rerio*). J. Am. Assoc. Lab. Anim. Sci..

[46] Schroeder P.G., Sneddon L.U. (2017). Exploring the efficacy of immersion analgesics in zebrafish using an integrative approach. Appl. Anim. Behav. Sci..

[47] Huang W.-C., Hsieh Y.-S., Chen I.-H., Wang C.-H., Chang H.-W., Yang C.-C., Ku T.-H., Yeh S.-R., Chuang Y.-J. (2010). Combined Use of MS-222 (Tricaine) and Isoflurane Extends Anesthesia Time and Minimizes Cardiac Rhythm Side Effects in Adult Zebrafish. Zebrafish.

[48] Chambel J., Pinho R., Sousa R., Ferreira T., Baptista T., Severiano V., Mendes S., Pedrosa R. (2013). The efficacy of MS-222 as anaesthetic agent in four freshwater aquarium fish species. Aquac. Res..

[49] Chen K., Wang C.-Q., Fan Y.-Q., Xie Y.-S., Yin Z.-F., Xu Z.-J., Zhang H.-L., Cao J.-T., Han Z.-H., Wang Y. (2014). The Evaluation of Rapid Cooling as an Anesthetic Method for the Zebrafish. Zebrafish.

[50] Amend D.F., Goven B.A., Elliot D.G. (1982). Etomidate: Effective Dosages for a New Fish Anesthetic. Trans. Am. Fish. Soc..

[51] Martins T., Diniz E., Félix L.M., Antunes L. (2018). Evaluation of anaesthetic protocols for laboratory adult zebrafish (*Danio rerio*). PLoS ONE.

[52] Martins T., Valentim A., Pereira N.M., Antunes L.M. (2016). Anaesthesia and analgesia in laboratory adult zebrafish: A question of refinement. Lab. Anim..

[53] Sneddon L.U. (2012). Clinical Anesthesia and Analgesia in Fish. J. Exot. Pet Med..

[54] Zahl I.H., Kiessling A., Samuelsen O.B., Olsen R.E. (2010). Anesthesia induces stress in Atlantic salmon (*Salmo salar*), Atlantic cod (*Gadus morhua*) and Atlantic halibut (*Hippoglossus hippoglossus*). Fish Physiol. Biochem..

[55] Blessing J.J., Marshall J.C., Balcombe S. (2010). Humane killing of fishes for scientific research: A comparison of two methods. J. Fish Biol..

[56] Pounder K.C., Mitchell J.L., Thomson J.S., Pottinger T., Sneddon L. (2018). Physiological and behavioural evaluation of common anaesthesia practices in the rainbow trout. Appl. Anim. Behav. Sci..

[57] Fagerberg J.H., Al-Tikriti Y., Ragnarsson G., Bergström C.A. (2012). Ethanol Effects on Apparent Solubility of Poorly Soluble Drugs in Simulated Intestinal Fluid. Mol. Pharm..

[58] Pittman J.T., Ichikawa K.M. (2013). iPhone^®^ applications as versatile video tracking tools to analyze behavior in zebrafish (*Danio rerio*). Pharmacol. Biochem. Behav..

[59] Pittman J.T., Lott C.S. (2014). Startle response memory and hippocampal changes in adult zebrafish pharmacologically-induced to exhibit anxiety/depression-like behaviors. Physiol. Behav..

[60] Mathur P., Berberoglu M.A., Guo S. (2011). Preference for ethanol in zebrafish following a single exposure. Behav. Brain Res..

