# Supplementary material for: Screening of Anaesthetics in Adult Zebrafish (Danio rerio) for the Induction of Euthanasia by Overdose
Source: Biology (Basel). 2021 Nov 4;10(11):1133. doi: 10.3390/biology10111133 (PMC8614824; doi:10.3390/biology10111133)
Supplement: Supplementary file 1 [file biology-10-01133-s001.zip › biology-1410533-supplementary.pdf]

Supplementary Material

Pages 2-3: Supplementary tables

Table S1: Page 2

Table S2: Page 2

Table S3: Page 3

Table S4: Page 3

Table S5: Page 3

Pages 4-9: Supplementary figures

Figure S1: Page 4

Figure S2: Page 5

Figure S3: Page 6

Figure S4: Page 7

Figure S5: Page 8

Figure S6: Page 9

Figure S7: Page 10

Table S1. Number of fish per batch (Experiment 1)

Batch no. \ Chemical	Isoeugenol	Clove Oil	2-PE	Tricaine [a]	Tricaine [b]	Benzocaine	Lidocaine HCl	Etomidate
**1**	5	5	3	4	4	5	5	4
**2**	5	5	3	3	3	10	3	11
**3**	5	5	3	6	4	6	4	13
**4**	12	13	3	5	4	9	3	12
**5**	3	2	3	4	2	3	3	14
**6**	3	3	4	5	4	7	4	—
**7**	4	4	2	4	3	5	3	—
**8**	9	9	1	6	3	3	3	—
**9**	6	6	4	6	3	4	2	—
**10**	—	—	5	9	3	—	3	—
**11**	—	—	3	—	4	—	4	—
**12**	—	—	3	—	3	—	4	—
**13**	—	—	4	—	3	—	2	—
**14**	—	—	4	—	3	—	3	—
**15**	—	—	4	—	3	—	3	—
**16**	—	—	3	—	3	—	3	—
**Total no. batches**	**9**	**9**	**16**	**10**	**16**	**9**	**16**	**5**
**Total no. fish**	**52**	**52**	**52**	**52**	**52**	**52**	**52**	**54**

[a] = 0.5 g/L. [b] = 1 g/L.

Table S2. Number of fish per batch (Experiment 2)

Batch no. \ Chemical	Benzocaine A	Benzocaine B	Lidocaine HCl A	Lidocaine HCl B
**1**	121	23	5	15
**2**	14	74	15	76
**3**	26	22	18	18
**4**	—	24	68	19
**5**	—	—	19	—
**6**	—	—	18	—
**7**	—	—	14	—
**8**	—	—	8	—
**Total no. batches**	**3**	**4**	**8**	**4**
**Total no. fish**	**161**	**143**	**165**	**128**

Table S3. Number of fish per batch (Experiment 4)

Batch no. \ Buffer	Tris (0.2 mL/L)	Tris (2 mL/L)	Tris (4 mL/L)	$NaHCO_3$ (1 g/L)	$NaHCO_3$ (2 g/L)	$NaHCO_3$ (3 g/L)	$NaHCO_3$ (2 g/L) + 50 mL/L EtOH
**1**	5	3	5	5	4	3	5
**2**	5	3	3	4	4	3	4
**3**	—	4	3	4	5	3	4
**4**	—	4	3	5	5	6	2
**5**	—	5	3	5	3	2	5
**6**	—	6	1	3	6	4	3
**7**	—	6	6	3	3	4	5
**8**	—	3	6	3	6	5	5
**9**	—	3	6	6	6	5	2
**10**	—	3	5	—	3	2	4
**11**	—	5	—	—	3	—	3
**12**	—	5	—	—	3	—	3
**13**	—	5	—	—	—	—	3
**14**	—	2	—	—	—	—	—
**Total no. batches**	**2**	**14**	**10**	**9**	**12**	**10**	**13**
**Total no. fish**	**10**	**57**	**41**	**38**	**51**	**37**	**48**

Table S4. Behaviour in fish after exposure to 1 g/L lidocaine HCl supplied with different buffers, numbers in percent (Experiment 4)

Behaviour \ Buffer	Tris (0.2 mL/L)	Tris (2 mL/L)	Tris (4 mL/L)	$NaHCO_3$ (1 g/L)	$NaHCO_3$ (2 g/L)	$NaHCO_3$ (3 g/L)	$NaHCO_3$ (2 g/L) + 50 mL/L EtOH
**Neutral**	40	19	7	24	14	19	25
**Husbandry-like**	40	68	71	74	76	73	73
**Aversive**	20	12	22	3	10	8	2

Table S5. Average time (in seconds) to loss of reflexes in first vs second half of experimental subjects (Experiment 1)

	Isoeugenol	Clove Oil	2-PE	Tricaine [a]	Tricaine [b]	Benzocaine	Lidocaine HCl	Etomidate*
Startle reflex:								
Average; first 26 (27*) fish	159	128	66	98	65	35	44	458
Average; last 26 (27*) fish	68	78	100	175	77	30	39	444
Opercular beat:								
Average; first 26 (27*) fish	185	88	55	103	38	35	35	30
Average; last 26 (27*) fish	233	60	72	140	70	30	31	31

[a] = 0.5 g/L. [b] = 1 g/L.

Experiment 1:

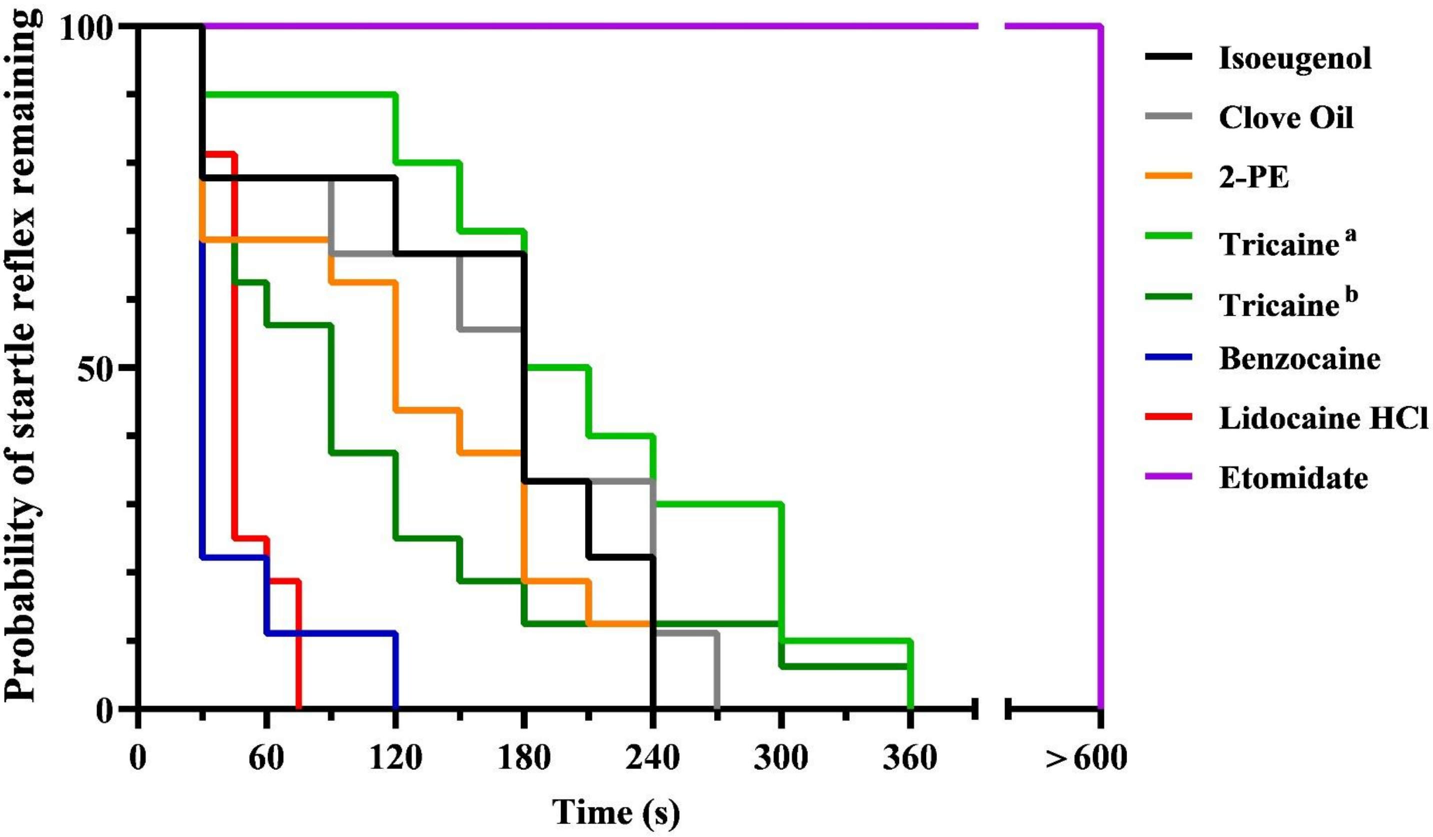

Figure S1. Probability of remaining startle reflex after immersion in experimental solution. The plots are based on batch results, where the singular fish within each batch that displayed the reflex for the longest time determined the value for the whole batch (*n* = 5-16, depending on test group, see Table S1 for details). [a] = 0.5 g/L. [b] = 1 g/L.

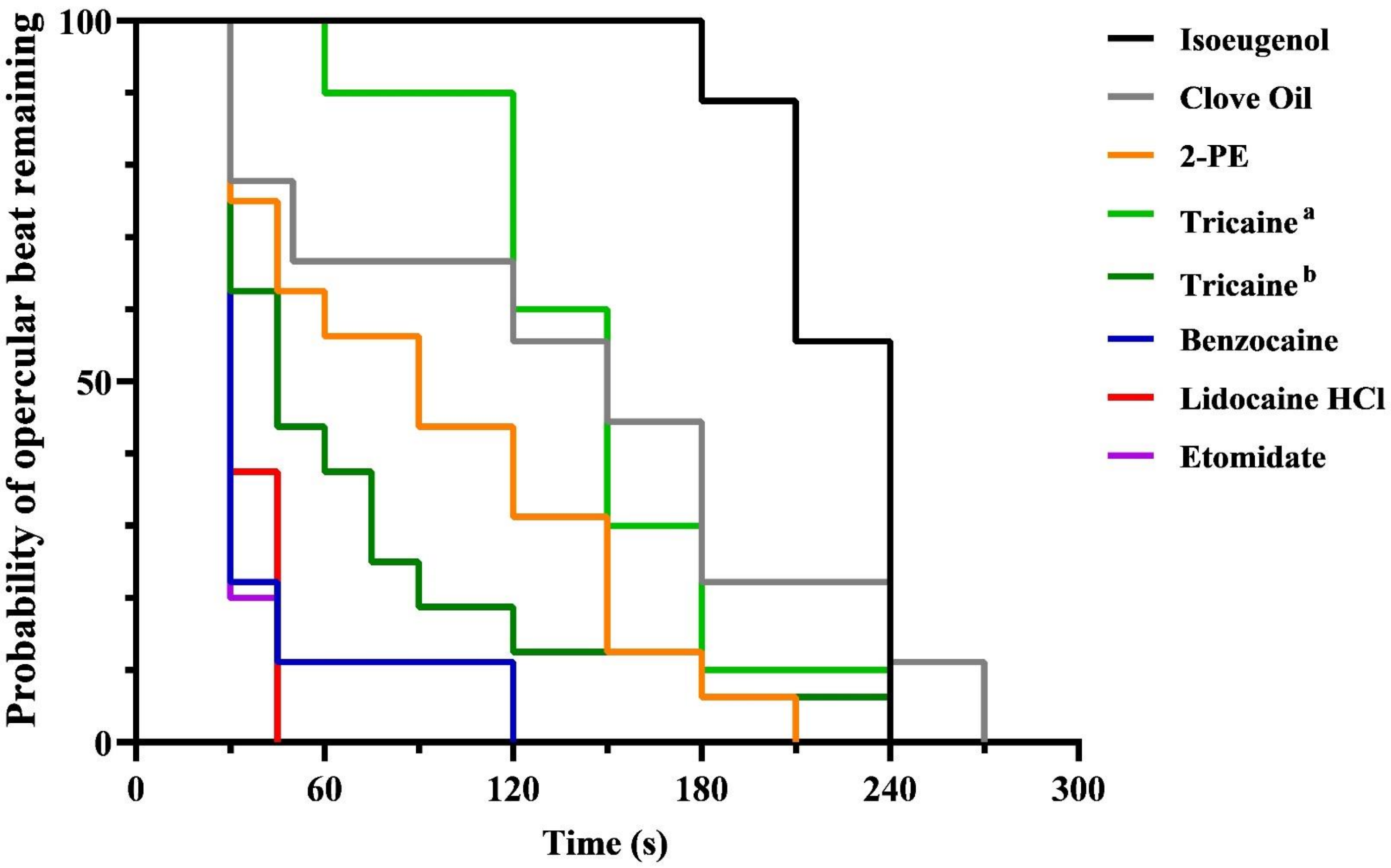

Figure S2. Kaplan-Meier plot describing the probability of remaining opercular beat after immersion in experimental solution. The plots are based on batch results, where the singular fish within each batch that displayed the reflex for the longest time determined the value for the whole batch (*n* = 5-16, depending on test group, see Table S1 for details). [a] = 0.5 g/L. [b] = 1 g/L.

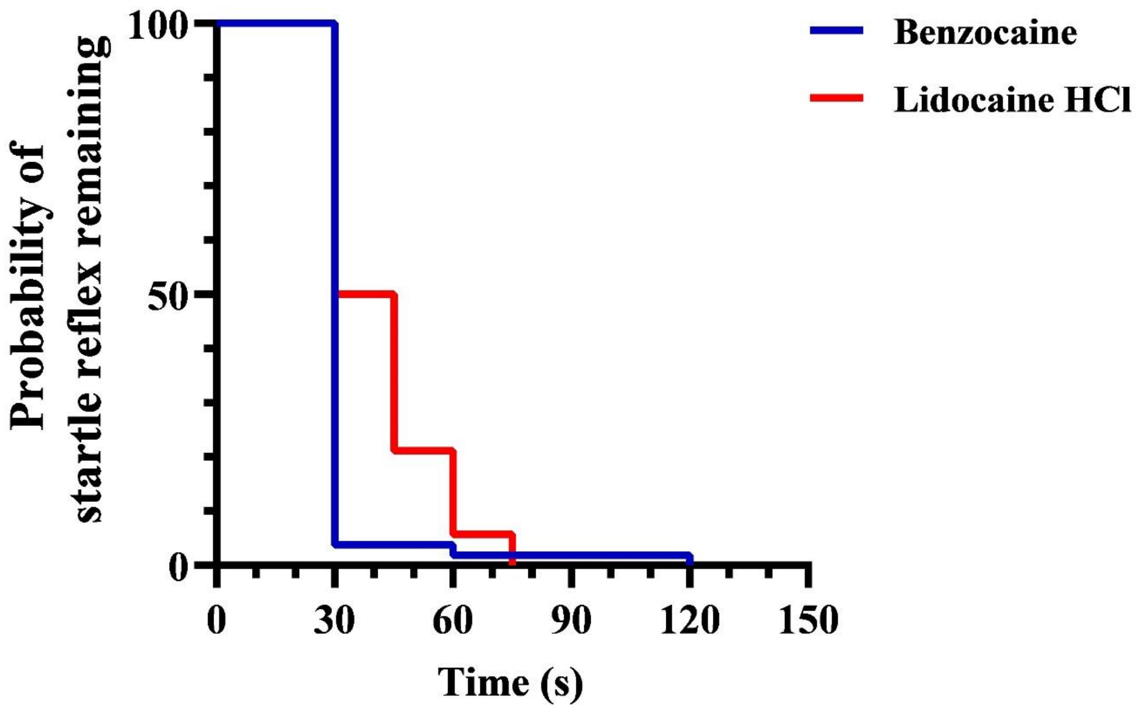

Figure S3. Time to loss of startle reflex in adult zebrafish after immersion in 1 g/L benzocaine or 1 g/L lidocaine HCl (+ 2 g/L $NaHCO_3$), measured at 15 s intervals until 90 s, then every 30 s until 300 s after exposure. Data are presented as a Kaplan-Meier plot describing the probability of fish having intact startle reflex over time (*n* = 52, see table S1 for details).

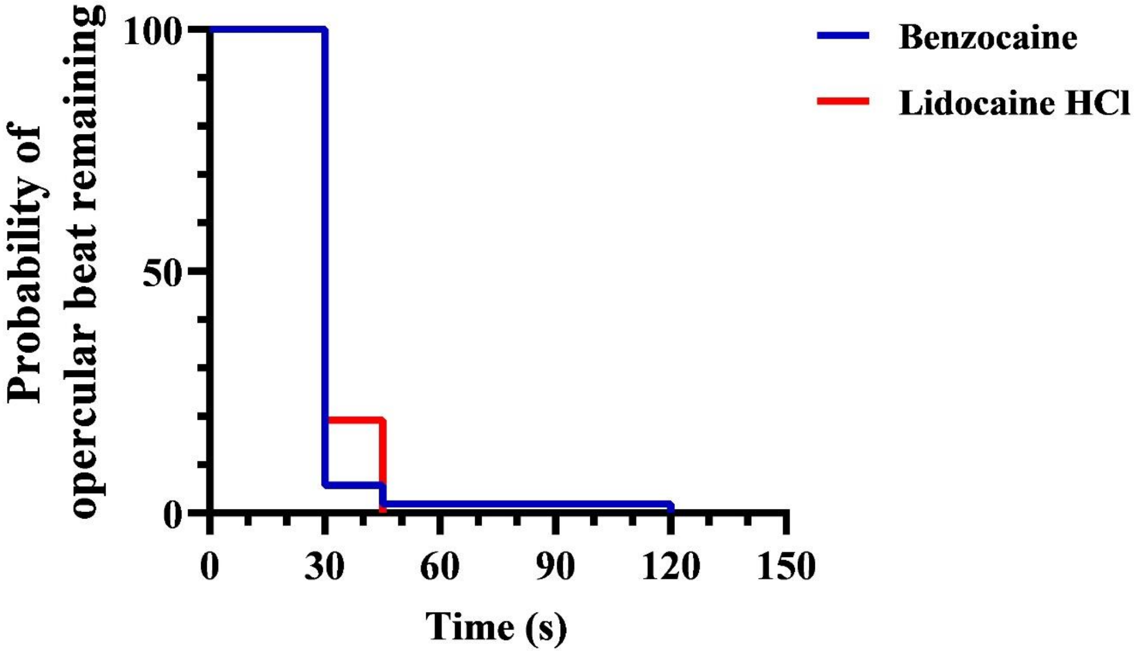

Figure S4. Time to cessation of opercular movements in adult zebrafish after immersion in 1 g/L benzocaine or 1 g/L lidocaine HCl (+ 2 g/L $NaHCO_3$), measured at 15 s intervals until 90 s, then every 30 s until 300 s after exposure. Data are presented as a Kaplan-Meier plot describing the probability of fish maintaining the opercular beats over time (*n* = 52, see table S1 for details).

Experiment 2:

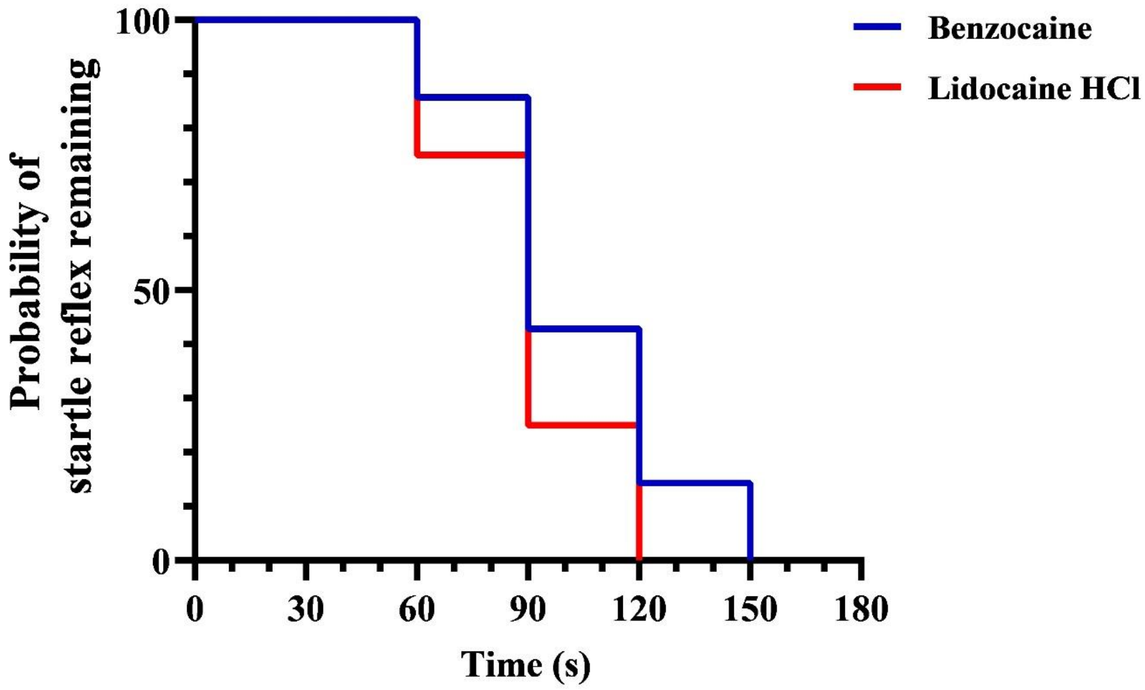

Figure S5. Probability of remaining startle reflex after exposure to benzocaine or lidocaine in mass testing. The plots are based on batch results, where the singular fish within each batch that displayed the reflex for the longest time determined the value for the whole batch (n = 7 and 12, respectively, see table S2 for details). No statistical differences were detected between treatments (log-rank test).

Experiment 4:

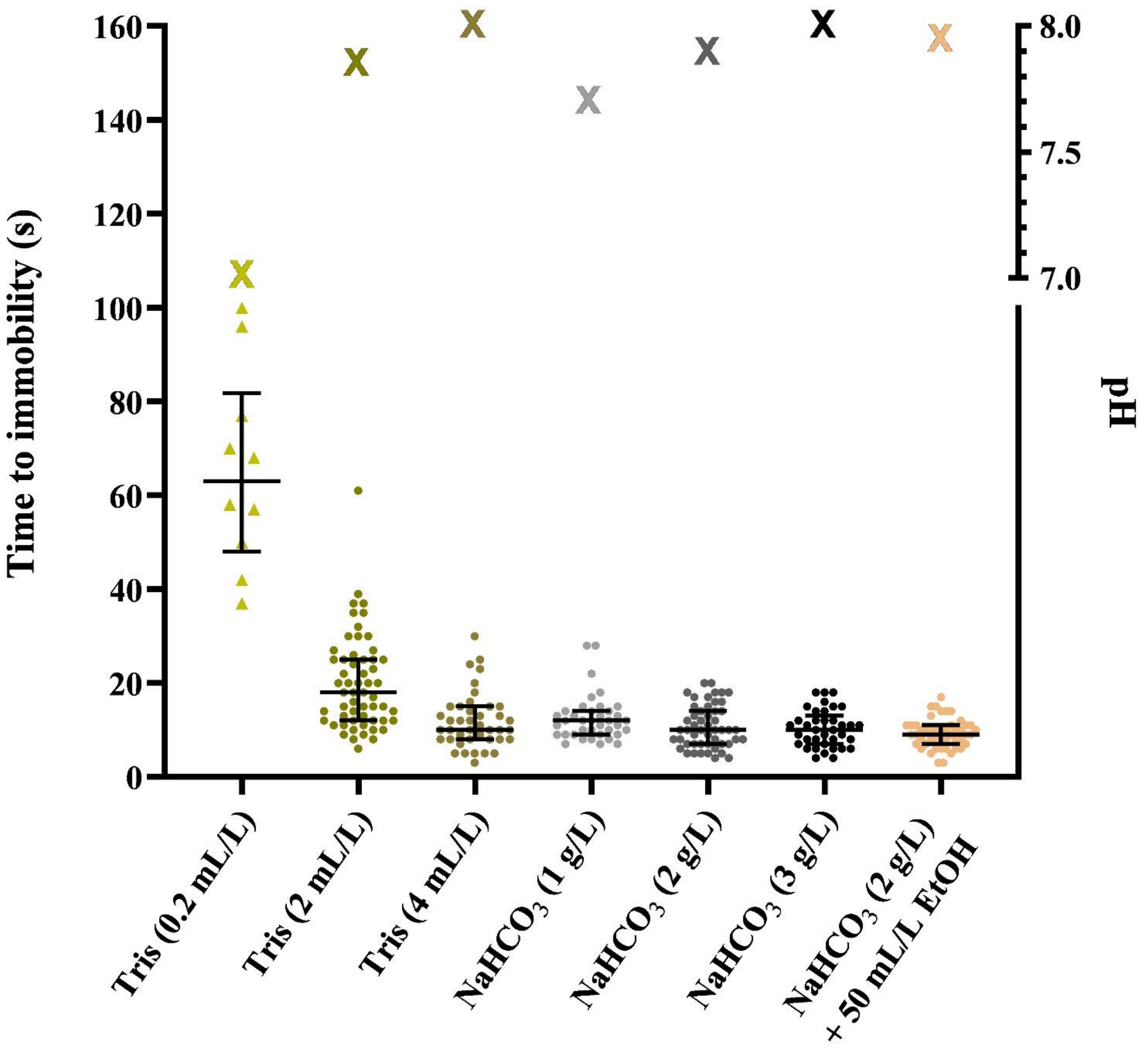

Figure S6. Time to immobility (in seconds; left y-axis) in adult zebrafish after immersion in lidocaine HCl supplemented with various buffers (*n* = 37-57 per treatment, see table S3 for details). Medians with interquartile ranges are indicated. Crosses mark the pH of the individual solutions (right y-axis). Note that Tris 0.2 mL/L treatments (marked with triangles) were omitted after first round testing due to long induction time (*n* = 10).

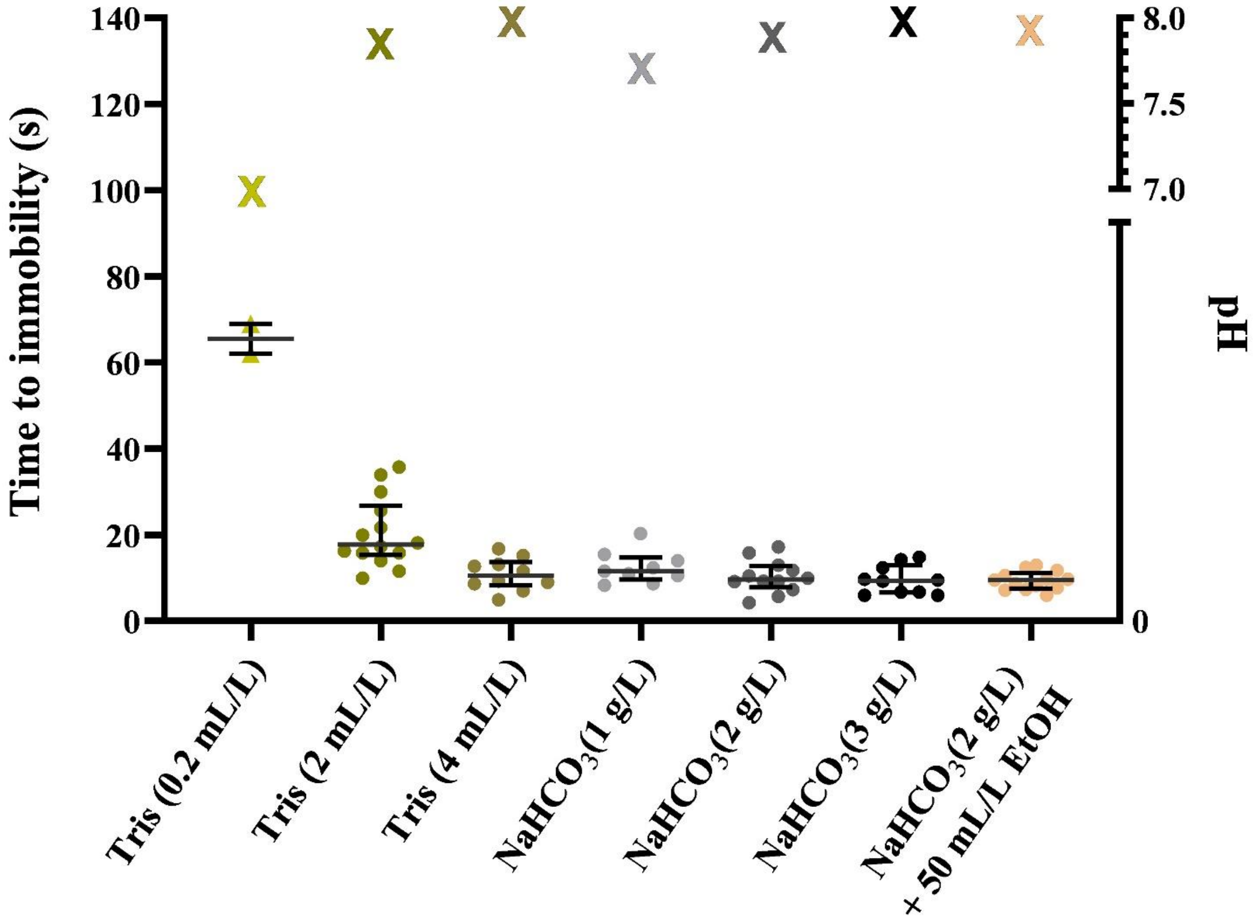

Figure S7. Time to immobility (in seconds; left y-axis) in adult zebrafish after immersion in lidocaine HCl supplemented with various buffers. The data are based on batch results, where the singular fish within each batch that remained mobile for the longest time determined the value for the whole batch ($n = 9$-14, depending on test group, see Table S3 for details). Medians with interquartile ranges are indicated. Crosses mark the pH of the individual solutions (right y-axis). Note that Tris 0.2 mL/L treatments (marked with triangles) were omitted after first round testing due to long induction time ($n = 2$).
